# The Book of Analysis; or a New Method of Experience, &c. &c.

**Published:** 1832-01-01

**Authors:** 


### THE BOOK OF ANALYSIS, OR A NEW METHOD OF EXPERIENCE; WHEREBY THE INDUCTION OF THE NOVUM ORGANON IS MADE EASY, &c. By *T. J. Todd*, M.D. London, 1831, pp. 186.

To those, who have made themselves intimate with the medical literature of the last thirty years, and have traced with care the progress of its advancement in some departments and the causes of its decline in others, nothing can have appeared more amazing than the unphilosophical, unconcocted trash, which has been teeming in volumes of every size from the medical press of this country. While it is truly curious to analyze the present spirit of authorship it is most dejecting and unconsolatory to witness its effects. A few observations most hurriedly made—a few fancies most rudely arranged—or if you will, a few facts carefully collated are laid down as the frame-work of a momentous publication in which general systems are dissected and condemned, preceding authorities are swept into annihilation by broad and dogmatic assertions, and a new creed is proposed for adoption which contests not only its superiority over all that has gone before, but its perfect adaptation for all future ages, nations and constitutions. A few rare and interesting specimens of disease are witnessed by the young practitioner, either in the wards of the hospital in which he received the elements of his education, or during the first months of his professional career;—these are embellished with imposing speculations, enriched by marginal references, loaded with a learned appendix, prefaced with a pompous dedication, and, emblazoned with a title-page, which promises every thing but what is to be discovered in the text. This essay—thus got up—is advertised into being by some interested publishers—puffed into notice by a few hireling scribes—sold and circulated—read and quoted—referred to as authority on points of faith, and ranked among the *monumenta laborisqu' ingenii* of the age, until its baseless pretensions are laid bare by the discoveries of subsequent writers, and all its facts and fancies are unsettled and unsaid.

Far be it from us to assert that this sketch of *book making* is universally, or even generally applicable. There are many to whom this censure will not attach, and we do most devoutly look forward to a speedy and large increase of their number. But it is undeniable, that very many of our present publications are scarcely worth the warehouse-material of which they are composed, and that not a few of them are positively injurious. False facts and distempered fancies are thus put forward to the world, which those, who cannot contradict, too frequently believe; while their refutation occupies the time of such as they are unable to convince. The remedy, which the author now before us would apply to all these grievances, is a rigid course of *inductive analysis.*

"The means, which my views of the improvement of medicine embrace, are neither many nor complicated. They may be comprehended in a few words—true, distinct, circumstantial observation—clear, severe, searching analysis." 118.

"I feel myself fully warranted, by long and ample experience, in affirming, that, whether it be considered in its scientific relations, or in its practical details, no department of knowledge so urgently demands the wholesome reform of a close and scrutinising induction.

Although the practice of medicine must be admitted to be unquestionably one of the most difficult of all the arts, it is still no easy matter to answer the question, which may very fairly be asked, why the art of physick, one of daily necessity and of daily exercise, should have improved so slowly, and been so devious and unsteady in its progress, and that after a written experience of more than two thousand years, it should still remain an instrument of such doubtful and uncertain application. I am by no means of opinion that the complex nature of the subject* which forms the business of medical research and of medical operation, furnishes a satisfactory explanation of this anomaly. The phenomena of health and of disease, the effects of food, of remedies, and of every agent capable of exerting an influence upon the living body, are objects of simple observation, are as cognizable as any other class of facts, and as certainly subject to a regular order as any other series of events. No one, arguing only from the past, has a right to say that this order is beyond the reach of observation, or that it is impossible to establish certain fixed principles concerning the manner in which these phenomena and these effects have place. And, consequently, no one has a right to deny that it may yet be possible to establish corresponding principles concerning the manner of producing these phenomena by art, of preventing them from taking place, or 'in quantum fert mortalium sors,' of arresting their progress. If these propositions, which are the general and compressed argument of Cabanis, be fairly put, it follows, that the cause of the uncertainty of medicine is not so much to be sought for in the difficulty of the subject as in the manner of observing and of studying it." 108.

We most cordially acquiesce with these sentiments, and we wish the profession could be made to feel their justice as acutely as they come home upon our own minds. With one part of it, we are grieved to believe, that it has become quite fashionable to designate medicine the science of chances, and to treat it in their own practice as an empirical humbug, which rests its reputation upon popular credulity. They make a boast of the uncertainty of physic, of the mysteries of disease and of the mummeries of practice! But such men are a disgrace to medicine. They are empirics, because they treat it as empiricism—they are grossly ignorant, because they will not stoop to the labor of making themselves acquainted with its resources—they neither know the extent to which disease is curable, nor the power which drugs possess, because they have entered their profession not as a science but as a trade; and because, forsooth, they kill their patients as often as they cure them, and are disappointed with the operation of medicines as frequently as they experience their efficacy, in consequence of this their own voluntary, gross and unpardonable ignorance, medicine must be held to be all a farce—a fable—a useless and unscientific imposition! But let us caution these slanderers of the healing art against conduct so unworthy. Let us tell them that not only is the deprecated and exaggerated uncertainty of physic, to a very great degree the effect of their own gross ignorance; but that if ever medicine shall become the thing they would seem to wish, it will only happen through such base conduct as they are guilty of. Let

---

* "Subjectum istud *medicinæ* (corpus nimirùm humanum) ex omnibus quæ natura procreavit, maximè est capax remedii, et vicissim illud remedium maximè est obnoxium errori. Eadem namque subjecti subtilitas et varietas, ut magnam medendi facultatem præbet, sic magnam etiam aberrandi facilitatem. Quo circa quem admodum ars ista (quo præsertim nunc habetur modo) inter præcipuè conjecturales; ita inquisitio ejus reponenda est inter summè arduas et accuratas."

them look to every name on the long list of the Æsculapian line, and they will find that the calumniators and cavillers of the faculty have invariably been the most uninformed, as well as the most unsuccessful; while patient industry was rewarded by the general improvement of the science, and the most splendid talents were impressed with a lively sense of its importance. Are we to be told that medicine can do nothing, because such as are ignorant of it can do nothing with it; or that it never will do any thing, because those, upon whom its progress depends, shamefully neglect it? In no instance that occurs to our recollection has any one branch of the healing art been perseveringly and judiciously cultivated, without recompensing the enquirer; and whenever we feel any tendency to such unreasonable scepticism as we are now reprobating, the very consciousness of such a state of mind should be taken as a proof, either of our being naturally disqualified for the profession, or of our having rendered ourselves such by negligence and misconduct. Let us not be understood as meaning that there is no uncertainty in medicine, no obscurity in disease, and no difficulty in attaining a thorough knowledge of the genuine principles of the healing art. Experience forbids such a supposition; but we do mean that much of the uncertainty, obscurity and difficulty, which is encountered, has arisen and continues to flow from the irrational manner in which medicine has been taught and studied. This no one can deny, who is capable of forming any opinion upon the matter; and, therefore, it is with wretched taste that those men, who vilify medicine as empirical, stamp upon it such a character, seeing that they themselves are the very men, to whom it is mainly indebted for whatever empiricism may attach to it. General inferences are drawn from particular premises, individual cases are made the models of entire epidemics, effects are confounded with causes and causes with effects, the sequent with the antecedent and the antecedent with the sequent,resemblances are discovered where none exist, and points of difference are detected where more minute inquiry could have found nothing but accordance. We write ourselves into practice, and when we have got it, the conviction of our having written on matters we did not understand makes us endeavour to explain away the errors our inexperience has committed. Thus are the more matured periods of our lives spent in rectifying the blunders of our earlier years, and is the profession, which we entered for gain, rested to satisfy our pecuniary necessities at the expense of every thing which can render it desirable.

"Medicine (Dr. Todd justly remarks) is not a science of experience in the same sense as the other sciences, for no other reason than because the instrument and the art of experience have never yet been applied to it. Were it possible to place medical facts within the grasp of induction, medicine must necessarily stand on the same footing as any other science of observation, perhaps of experiment. The materials of experience are not wanting. The stores are ample and well-furnished. Every day is busy in producing more, and perhaps of a better kind. There is nothing intrinsically in the nature of the facts which are of the province of medicine which prevents them from being analysed and reduced to order, and from being thus made to contribute more or less to the common stock of experience. The analysis of medical observations is no doubt beset with some difficulties which are only to be overcome by the united labours of many, and by repeated efforts; but if this important work was only undertaken in a right spirit, and steadily persevered in—if the method of observing was reformed, if the observations were subjected to the assay of a searching induction, it is impossible to foretel what might be the happy results; and certainly not before such a trial has been fairly made, is it allowable to say, that medicine cannot be elevated to the rank of the other sciences. This is no new view of the matter, no enthusiastic anticipation, the offspring of a new theory. It is the opinion of Sydenham* and Baglivi,† equally disciples of Hippocrates and of Bacon, and the two most illustrious names, who, since the revival of letters, have honoured and adorned our art." 113.

Many, we believe, permit themselves to be offended with their profession by expecting it to do more than it promises, and by thus meeting with disappointment. They forget that it is not an abstract science—that its principles neither require, nor admit of demonstration—that the machinery by which and the materials upon which it works are of such a nature, as to preclude the very possibility of unerring, uniform and strictly mathematical results. The same disease is modified by a thousand circumstances, over which we have no control; the same constitution varies from causes which we cannot explain; and the same medicine depends for sameness of operation upon so many fickle contingencies that, without giving rise to any paradox or meriting any blame, it may produce almost opposite effects upon the same system under different states of constitution. These undeniable facts are wholly lost sight of by our imperious lawyers and our critics of the broad sheet, when the character of medicine, or the reputation of the faculty becomes the subject of animadversion. If all the questions, which in their wisdom they think proper to propose, cannot be unqualifiedly answered—if we cannot lay our rule and plumb-line to every circumstance and statement, medicine is called an unsatisfactory science, and medical men are ridiculed in open court. Truly, many of those, whom we have seen in witness-boxes, well merited the legal castigation which they met with; and, while we abhorred the impudence and coxcombery of the powdered wig, we could not help subscribing our name in full to the justice of the punishment. But it should be known by men, who thus step out of their own professional province to take such public liberties with those who ought to be, if they are not, their entire equals both in talent and attainment, that the very essence of our science is obnoxious to variation, and that those results, upon which we can most safely depend as being the most carefully arrived at, are at the very

* "Si vel unus tantum per singula mundi secula hoc modo unicum tractaverit morbum, medendi ars, (quæ medicorum est provincia,) à multis retro annis ad ἀκμὴν pervenisset, *omnibus absoluta numeris*, saltem in quantum fert mortalium sors."

† "Enim vero dum tota medicinæ prudentia in eo posita videatur, ut morbum cum morbo, tempus cum tempore, hominem cum homine compares, quo advenientia, et crescentia mala suis certis signis, ac nominibus ea distinguere, iisdemque idonea aptave remedia adhibere queas, nemo certè inficias iverit, nullam prorsus, et nobilissimæ scientiæ exornandæ, et curandis hominibus utiliorem operam navari posse, quam si præstantissimæ artis studiosi, immenso retro tempore jam factas a majoribus nostris observationes attenderent, novasque in dies animadverterent ac notarent. Quam quidem promovendæ artis rationem, si jam inde ab Hippocratis temporibus ad hanc nostram usque ætatem constanter homines retinuissent, dici vix potest, quot quantique progressus hac hodie parte haberentur. Cum vero rem alioqui adeo necessariam, tam præclaram, atque ita feliciter institutam reliquerint, ut se infinitis et (ut Apostoli verbis utar) *interminatis quæstionibus*, λογομαχιαις implicarent, aliam afferre causam non possem, quam offensi, ac ulsciscentis Numinis iram."

best in general only little better than near approximations to abstract truth. It must be as palpable to every one in the profession, as to those without its pale, that it is becoming quite the *ton* to laugh both at physic and physicians. Scarcely is there a newspaper, in which may not be found sneers, insinuations, or direct charges against us, either for carelessness or ignorance, stupidity or selfishness; and although the penalty we are made to pay is probably beyond our crime, all this abuse can be traced with ease to our own conduct. Were we less easily led away by names instead of things, by modes instead of entities, by probabilities instead of certainties, by authorities instead of facts; did we observe attentively before we decide positively, did we experiment generally before we conclude universally, did we theorize less, philosophize more, write only what we know, and know only what we have good reason to believe, we would, in our opinion, soon become less the prey of empirical impostors on the one hand, and less the butt of forensic raillery on the other. Nothing can be more subversive of all good than a hasty and uneducated spirit of inference, which conjectures truth to save trouble, and hazards the merest fancy of the imagination rather than labour out the demonstration of a practical problem by an unwearied scrutiny of disease; and, therefore, nothing can be more conducive to obviate all these pernicious practices than an unyielding obedience to rigid, inductive experience.

We have our own misgivings, notwithstanding, as to the unaided sufficiency of this remedy in rectifying so many vices. But that all may be equally capable of judging with ourselves, we shall lay down a hasty outline of the Doctor's remedial plan, with this necessary remark, by way of preface, that our sketch will be confined to the bare principles of his system, sending the reader who seeks for further information, to the *Book of Analysis* itself. The Doctor recommends a series of tables to be constructed according to the different subordinate intentions, which they are designed to accomplish. By means of these tables all the circumstances, connected with any particular facts or instances, are classified by signs or hieroglyphics, according to their points of resemblance or dissimilarity. Each instance or case is noted down and expressed by some letter of the alphabet, which is its sign; and each circumstance is written down in full by itself in a separate column. The signs of the instances are arranged in their respective columns in the following manner—

"Taking A to represent the first, B, the second, and so forth; as the circumstances of the first instance were written down in the broad column, or *column of circumstances*, (in the order in which they presented themselves,) the sign A was placed opposite to each, in the column of the first instance. In the same manner, in the second instance, for every circumstance which had already been found in the first instance, A was placed in the column of the second instance. Such circumstances as had not been found in the first instance, were added to the column of circumstances, and the sign B was placed against them, in the column of the second instance. In like manner, in the third instance, the circumstances, the same as those of the two preceding instances, were denoted in the column of the third instance by their signs, A or B, respectively; and such circumstances of the third instance as were not found in either of the preceding, being first noted down in the column of circumstances, were denoted by a new sign, *viz.* that of the instance C. And so on in all succeeding instances, the same circumstances recurring continued to be denoted by their original sign, and every new circumstance, being first inserted in the column of circumstances, by the sign of the instance which it first occurred." 25.

It is thus obvious that the relations of the signs indicate those of the cirumstances, and that, by ascertaining the degree and constancy of these relations, the relative position of every circumstance—whether cause or effect, the effects of a common cause, or the cause of a common effect—is accurately ascertained. In a third column these signs are brought out of the column of instances, and arranging all the same signs together, each is placed in its own vertical line. In this way all the circumstances common to all the instances, however numerous and complicated they may be, are adjusted under their common sign; and this part of the Doctor's process is called *classification by affirmative circumstances*. But it is not difficult to see that this plan of classifying does not only indicate the relationship, which subsists between all the circumstances placed under the same sign;—it also denotes the *absence* of relationship between different signs, since each succeeding class of circumstances is excluded by its predecessors.

"Thus the class A of circumstances having been found present when the class B of circumstances was absent, according to the principle* laid down by Lord Bacon, the class A excludes the class B.† In the same manner, the classes A and B exclude the class C, and so on, the other classes successively. Therefore, as it is the effect of this process of classification to bring together under the same sign all those circumstances which have co-existed, so is it to exclude from each other, and to distinguish by different signs, those which are independent of each other." 29.

This gives rise to another mode of arrangement, which he calls *classification by negative circumstances*, and which is attained in the following way—

"For any circumstance of the class found in any instance, the sign of the class must be placed opposite that instance in the column of the circumstance, and so on for all the succeeding instances. But when in an instance any circumstance belonging to a class is found defective, the sign of that instance, instead of the sign of the class, is to be placed in the column of the defective circumstance; and this defective circumstance must retain the same sign in all the subsequent instances. In this way, a second classification, and a second process of exclusion is established; for, whenever a circumstance of a class is found deficient, the sign of the class being replaced by the sign of the instance in which it is first found deficient, it becomes excluded from the class on the evidence of the instance or instances in which it may be found wanting. After the circumstances have been compared in this way, in all the different instances, and the signs placed accordingly, the whole classification must be corrected at the foot of the table, by substituting for the signs of the circumstances, those signs

* "Itaque naturæ facienda est prorsus solutio et separatio; non per ignem certe sed per mentem, tanquam per ignem divinum. Est itaque inductionis veræ opus primum (quatenus ad inveniendas formas) rejectio sive exclusio naturarum singularum, *quæ non inveniuntur in aliqua instantia, ubi natura data adest. Aut inveniuntur in aliqua instantia ubi natura data abest,* aut inveniuntur in aliqua instantia crescere cum natura data decrescat, aut decrescere cum natura data crescat."

† "To use the language of Lord Bacon, A is the representative of instances *absentiæ in proximo* of the circumstances designated by B."

which the circumstances have assumed in the particular instances; so that those circumstances only which retain the sign of the class, continue to belong to it." 33.

By means of these two forms of tables, which he calls *tabulæ inveniendi*, he determines the relations of circumstances to one another; but as it is not only desirable to ascertain these generally, but so minutely that all their degrees and modifications may be discovered, he places over the sign of any circumstance, under any degree or modification, the sign proper to the instance in which this degree is found; and

"In order to avoid a confusion of signs, the corresponding letter of the Greek alphabet, may be used instead of the Roman letter. Thus suppose a circumstance A recurs under a modification in the instance D, this will be expressed by Aδ. If therefore, the modification of the circumstance Aδ is of sufficient importance to induce any change in any other circumstance with which it may be in relation, there will of course be found other circumstances, in the same instance, with signs of modifications, or there will be some new combination of the signs, or some signs absent or present, out of the usual order. And therefore, where circumstances under the same signs do not, by their signs, express such mutual affections or modifications, they must be excluded from having any constancy of relation affirmed of each other." 37.

This method of induction may be easily extended to almost any number of instances, so that one table, or even a series of tables of signs may be compared with those of another, by reckoning the *number* of signs of each circumstance, or the *number* of times each circumstance recurs in the different instances, and by placing these numbers in figures under the proper signs in the column of classification. This numerical adjustment of the signs to the circumstances the Doctor has called *rectification of the signs*, and is a process of arrangement by which the signs, contained in any number of tables, may be accurately classified.

The Doctor's entire analytical scheme is skeletoned in the following passage.

"I. *The translation of circumstances into signs*, which express not only the presence and absence of circumstances relatively to each other, but also their reciprocal modes and modifications; and which standing precisely in the position of the circumstances, indicate all the possible relations which can exist between them.

II. *The classification of signs by affirmative circumstances* according to their conjunction in different instances or individual cases, by which those co-existing are classed together, and those not co-existing are excluded and separated from each other.

III. The extension of this principle to any number of instances by the *rectification of the signs*.

IV. *Exclusion* of those circumstances from the classes formed by the previous processes which are not found constantly present in the classes; 1st, by the *classification by negative circumstances*, and 2dly, by *deficiency of mutual variations and modifications*." And the *Rules for performing the different processes of this scheme* are thus laid down—"I. *Translation of circumstances into signs*. For every circumstance found in any instance, place a sign opposite to it in the column of the instance. If the circumstance has been already found in any previous instance, the sign by which it was first denoted must be repeated, but if not so, the sign of the instance will be the sign of the circumstance. If any circumstance already found in some previous instance, presents itself in another instance with some variation or modification, the Greek letter corresponding to the Roman letter, the sign of the instance, is to be placed over the sign of the circumstance, and this letter must continue the sign of the same modification in all succeeding instances.

II. *Classification by affirmative circumstances.* When all the circumstances have been translated into signs, according to the preceding rule, the signs of the circumstances are to be carried into the column of classification, each under their respective sign, so that the same signs may stand in the same vertical line; or, the co-efficient of each sign is to be placed in the column of classification instead of the sign.

III. *Rectification of the signs.* The circumstances and the signs by which they are represented in the column of classification, are to be transferred to their appropriate columns in the table of rectification. If a circumstance is represented by the same sign in all the classifications, the sum of its co-efficients is to be placed under that sign in the column of rectification. But if a circumstance be denoted by different signs in the different classifications, it is necessary to find that sign whose co-efficient carries the greatest sum proportionate to the number of instances, and the sum of the co-efficients of all the signs must be placed under that sign in the column of rectification.

IV. *Exclusion.* First, by the classification by negative circumstances, the sign of the instance is to be substituted for the sign of a class wherever any circumstance of a class is found defective; and, second, any circumstance of a class which does not indicate modifications or affections reciprocally with the other circumstances of the class, is to be rejected from the class.

V. The products of the process of induction are to be at last transferred to the table of *Synthesis*, where every circumstance standing under its proper sign, whether of class or sub-class, shows the relations in which they all stand to each other." 57.

Such, then, is the method of induction proposed by our author, with the view of obviating the injurious effects of the present disorderly and unphilosophical spirit of inquiry; and he believes that, if carefully practised, it will be productive of many important advantages—in directing the attention to *all* the attending circumstances of each instance or fact—in attaching the proper degree of attention to *each* attending circumstance—in suffering *nothing*, which may tend to increase our experience, to pass unnoticed—in enabling the mind to dwell upon the consideration of *any number* of circumstances—in reducing observation to the guidance of *definite* rules of admitted accuracy; and, finally, in establishing useful *habits* of close perception and clear arrangement. To facilitate, as much as possible, the application of this plan to the various subjects of inquiry for which it may be adapted, he has drawn out a very minute and elaborate set of tables, suited in construction to the different characters of the materials to be analyzed. This portion of his work he calls the *methodus cantabulandi;* and in the fifth, which is his last chapter, he makes an ingenious application of his tabular analysis to medicine—physiology—phrenology—animal magnetism—chemistry—meteorology—natural history—the evidence of testimony—statistics and political economy.

The reader is now as well prepared to decide upon the value and practicability of Dr. Todd's system as we are, and we cannot but apprehend that he will concur with us in thinking that, however just in its principles and ingenious in its application, it is infinitely too complicated and minute, at least for the purposes of medicine. If we cannot prevail upon eight-tenths of the profession to submit their minds to those pre-requisite ordeals—an ordinary acquaintance with their native tongue, and an ordinary degree of professional attainment—ordeals so preliminary and so pre-requisite to render this Baconian system of analysis and synthesis at all available—if we cannot persuade them, before entering the lecture-room, to prepare their minds for scientific study, by making themselves moderately familiar with English composition and classical instruction; and if we cannot induce them, before entering upon the awful responsibilities of actual practice, to lay down something like the groundwork of a good professional education, upon which future opportunity and future labour might construct more advanced attainment—we hold it utterly Utopian to hope that any zeal, or talent, or incitements, which we can display, will prevail on them to sit down, with the minds and habits they at present have, to the study of an abstract and complicated series of analytical tables, which it requires a fully matured, and not a very uncultivated intellect even to comprehend. Look at the disciplinary system of the present hour, which qualifies the general practitioner for entering upon the duties of active life. Is it a system, which can be expected for a moment to give to the mind that adequate degree of preparation, which could make the Doctor's inductive system of the slightest value? After standing behind his counter for *half a dozen years*, learning the colors and smells, weights and measures, tastes and prices of his drugs; and after devoting about *twice as many months* to an investigation of their sanative principles, and application to the treatment of disease—to the anatomy, functions and laws of the human frame—to the manifold accidents and diseases, to which it is obnoxious—to the characters of these affections during life and their pathology upon dissection—to midwifery, chemistry and jurisprudence—the candidate for professional honors goes then before his college with confident assurance, passes through the fiery furnace with flaming reputation, purchases a share in some established business, and sets himself down for life, the authorized and accomplished veteran of science! We again ask is this the curriculum, which can make him a philosopher? Is it by such few and hasty strides that he can traverse the distant space, which lies between the infancy of thought and keen, penetrating, inductive reasoning? The truth is, the mind requires no mean degree of cultivation, ere it can become a successful disciple of the Baconian school. Its powers must be expanded by exercise, they must be inured to fatigue, they must be drilled into perfect submission to the judgment, they must be educated to abstract thought, to acute observation, to careful inference. And it is only during the childhood of the mental powers, that all this can be accomplished. It is not during the hurry of an oppressive and anxious occupation; it is not when habits early formed have sealed up the thinking faculties to a certain course of operation, and have disqualified them by long abuse from close and continued induction, that the *novum organon* is to be laid before them. Such minds must travel along their own beaten path in their own accustomed step. We can perplex and confound them, much more easily than either regenerate, or rectify them. We are most agreeably mistaking both the average calibre of their intellects, and the average stock of their attainments, if many more than a half of our professional fraternity will comprehend even the plan, which Dr. Todd recommends them to employ in observation; and if so, what likelihood is there of such a plan being carried by such minds into general operation?

It is to our rising and future generations that we look for medical reform —genuine, fundamental reform—that reform of existing customs, and that improvement in the education of our youth, without which neither our charters nor our colleges, our corporate bodies or anomalous monopolies, can do us either much serious injury, or good. Medicine must be *taught* as a pure, inductive, experimental science, before it can be very generally *practised* as such. It is from our schools and our seminaries that analytical philosophy is to commence its career of medical reform; not from the shop of the druggist, or the chamber of sickness. Let the materials of our professional fabric be fashioned and fitted at the quarry ere they are worked up into the body of our building. It is not when they are grounded in the foundation, or cemented into the superstructure, or placed over us as corner-stones and coverings, that we are to chip and chisel them. All this should be done in the nursery, the grammar-school, the academy and the lecture-room. It is there that the book of analysis must be sent, that its principles must be explained, enforced and inculcated. It is much better adapted to develop the green and growing mind, than that which age has rendered stationary, and custom unchangeable in its habits.

But it still becomes a question with us of no mean interest, how far we should be doing wisely to recommend to the rising generation of medical practitioners all the details of the application of his inductive plan, which the author has thought it prudent to make to the advancement of true medical science. The Doctor admits that "an improved method of the study and, consequently, of the practice of medicine is only to be expected from an improved method of education," and, therefore, to this length we agree; but, in as far as he attaches much more importance to "the method of teaching than to the matter which is taught," we stand very widely at issue.

"My meaning (he observes) will be quite misunderstood, if it is supposed that I imagine, that such an object can in any degree be obtained by the additional studies which have of late years been imposed upon those qualifying themselves for the practice of the healing art, in all its different branches. No doubt such studies may contribute much to the general accomplishment of the physician, and, giving him a more entire view of nature, by enlarging his comprehension, may increase his resources. But these advantages, seldom felt by more than a few individuals happily gifted, are altogether subordinate to the principal object of medical education considered in relation to the many. My notions of the improvement of medical education are in every respect opposed to these alterations, and relate more to the method of teaching, than to the matter which is taught. It seems indeed to me by far more natural that the student should pursue the same course in acquiring the knowledge of his profession in the schools, which he must in after-life follow in his practice, and that the synthetical teaching from the chair should be replaced by analytical observation in the hospital. I cannot help thinking that instead of acquiring a superficial acquaintance of sciences, sometimes remote, generally irrelevant, and which do certainly in many instances tend to establish habits of the mind by no means favourable to the practical details of medicine, it would be a more advantageous employment of the period of study, if the pupil were instructed and exercised in the right use and application of the true method of observation, which in active life must be the guide and the instrument of his professional labours." 119.

No one can deny but metaphysics are indispensable to qualify the mind for the inductive philosophy. Where the sifting of appearances, the discernment of relations, the weighing of evidence, the discovery of hidden and abstract truth are cultivated and required, it were ludicrous, no doubt, to expect much from a mind that was left ignorant of its own structure, powers and resources. As well might we expect the mechanic to work successfully without his instrument, as the medical philosopher without a mind intimately versed in its own mechanism, and in the most successful methods of applying the agency of its varied faculties to the elucidation of subjects submitted for examination. But it appears to us that, after the mind has been most skilfully educated in the science of itself, as well as in that of strict analytical induction, the task of its tuition has been no more than half accomplished. It must be taught to direct its powers to the most useful objects. The observing principle must be led to the fountain-springs of observation, ere the inductive faculty, be it ever so acute or so capacious, can avail much. A false lesson at this period of its pupillage may absolutely ruin it, or even render it more a curse than a blessing to the interests of science. What avail the expense and splendour of its education, if the matter, which it is taught, be unimportant or, what is worse still, if much that it ought to learn be disparaged or concealed. Now, we cannot exculpate the Doctor from all blame in this respect. The first object unquestionably is, to educate the mind to observe, but the second, and by no means inferior object, is to teach it the most befitting subjects for observation; otherwise its time and talents may be devoted to objects wholly unworthy of them, while others may be neglected which should be understood. We totally disagree, therefore, with the author in supposing that the *matter*, which is taught, is of less importance than the *manner* of teaching it, or that the present system of teaching our youth, what he calls, "additional studies" is in the least degree calculated to retard that improvement, which it is his object to introduce. We do not wish to see any one branch of medical science disparaged. For the accomplished Baconian physician they are *all essentially* important, and most usefully available both to ornament him in society and to prosper him in practice. No doubt, some are of greater moment than others, and to such the mind should be applied with greater earnestness, and where all cannot be attended to, prudence will wisely select the most important; but, in our estimation, there is more danger of our youth being taught too little than too much. The following passages will shew what are some of the "additional studies" alluded to.

"I think it proper to explain that it is not from the cultivation of medicine in specialities and dismembered parts that I augur much good. Indeed I am not aware of any thing which has been more adverse to the diffusion of correct notions, and more opposed to the establishment of truth in medicine, than the division of its study into different branches, thus separating the consideration of the causes of disease from the phenomena which they present, and from the method of treating them." 115.

Again—

"I am therefore not of the number of those who expect that the power of medicine in controlling disease, is to be greatly increased or extended by *curative indications* deduced from the phenomena which the scalpel can disclose. These, at best, can present only a fraction of the data upon which must be founded any deduction for the successful treatment of a disease. If I do not much mistake, the assistance to be derived from the study of morbid anatomy, can be only indirect, and must be confined to the help it may afford towards individualising diseases, if I may so speak; for by demanding a closer observation of particular cases it may facilitate the application of specific methods of treatment to specific cases." 116.

Again—

"In like manner I have little hopes of the improvement of medicine by the exclusive study of therapeutics. Some physicians would have us think that the power of medicine is to be vastly enlarged by the discovery of specific remedies for particular diseases, or of new agents capable of modifying or removing diseased actions. But the experience of ages forbids us to indulge in these expectations." 117.

On the contrary—

"I am of opinion that the student having been thoroughly disciplined in *classical and mathematical* learning, *(quamvis non faciat medicum, aptiorem tamen medicinæ reddit,)* and having been prepared by an *adequate* acquaintance with anatomy, physiology, chemistry, and materia medica, should at once proceed to the hospital. It is there, I think, that this knowledge of diseases ought to commence, so that he might know nothing of their symptoms by name, before he has made himself familiar with the reality. The symptoms of disease should be first demonstrated to him on the living subject, after the manner of teaching natural history, and not until he has acquired a distinct notion of them and undertood them accurately, ought he to learn the names by which they are technically distinguished.*" 121.

There may be some of these positions, into the truth of which we might, peradventure, be argued; but there are some of them so totally *ultra* to every thing we have been accustomed to think rational, that the great Lord Bacon himself could not seduce us into their belief. Were we to select that "additional study," which tended the most and the soonest to prepare the practical physician to grapple with disease, we should chuse *pathology*; and yet, were it not for one of the above passages, it would scarcely be credited that an observer, so cautious and so circumstantial as the Doctor, never once hints at even the *propriety* of *occasionally* consulting morbid anatomy in a sketch which he has drawn of an *analytical hospital*, which, by the way, he seems to make the school-room and college, the alpha and omega of a practitioner's curriculum. We have heard some of our grey-haired fraternity, of the old school, grudge and grumble most exceedingly at the expense and labor which are now so generally given to the cultivation of pathology, and we are not prepared to deny that it may not, perhaps, occupy too disproportionately the attention of the present age; but we do think that the Doctor acts most inconsistently with himself, in recommending the rigid system of observation which he has laid down, and thus keeping in the back ground, if not entirely out of sight, what is very generally considered nearly an entire half of the very field of observation. Symptoms unquestionably

---

* "Les connoissances qu'on acquiert dans les écoles, ou dans les livres, ne peuvent donner ni cultiver la sagacité des sens :—Les vraies connoissances de notre art ne sont qu'un ensemble, plus ou moins complet, des sensation recuillies au lit des malades ; ces sensations ne peuveut être fournies, que par les objets mêmes qui les produisent. Ainsi la lecture, à proprement parler, ne nous enseigne, en quelque sorte, que ce que nous savons déja.—*Cabanis*."

must be watched—the living and breathing features of disease must most carefully be studied; but cases are occurring daily, during the management of which the most lettered symptomatist must feel himself wading in unfathomed waters, without a prop to support his *ratio medendi*, beyond loose analogies and ill-defined indications. Separate all we know of practical medicine through the agency of pathology, from that which we have learned by the aid of symptoms, and what an indigested mass of unsettled mixture would medicine be! The voice of symptoms is often inarticulate when audible, and not unfrequently inaudible when distinct. Besides, it is not symptoms that the intelligent practitioner bleeds and blisters, drugs and struggles with. He sees deeper. He looks through these outward signs to their cause and seat, their rise and relationship. But how is he to do this, without knowing the anatomy and watching the disorganization, which disease has wrought in internal organs in corresponding instances? And how can he achieve this, without minute, patient and habitual inspection of the dead? If he despise this internal and hidden light, he must make many a fateful step in utter darkeness; he must cure many a patient by a lucky chance; and he must lose many a life, without being able to say where lay the disease, what was its precise nature, how far had it advanced, was it certainly beyond the reach of medicine, or by what means might it have been possible to effect its cure. The author's arguments in favour of the inductive philosophy we feel and estimate; the tabular analysis which he proposes we consider highly ingenious, and if sufficiently practical very highly useful; but really it is somewhat *outré* to be told that mathematics are more essential to a physician than morbid anatomy! Simpson's Conic Sections, Bonnycastle's Algebra, and Lardner's Euclid stand a fair chance of pushing Baillie and Bright, Andral and Abercrombie off our shelves; and the problem of the three bodies, or the asses' bridge, threatens to usurp the rank of universal science!

We are far from wishing to be, or even appear severe. Dr. Todd's book displays too much sound thought and strong judgment, to permit any straggling error to seduce us out of the respect which we owe to a man of talent, labouring in his own way, for the improvement of his profession. But at the same time, it is due both to the profession and to ourselves, as pathological practitioners, and as disagreeing most irreconcilably with the Doctor on this very fundamental point, to show wherein and how far he appears unorthodox; convinced that, however we may wound a favourite crotchet, we must, in all candour, warn our readers against the adoption of such a heresy. We have read Dr. Todd's Book of Analysis with pleasure—we cannot speak in terms too flattering of the spirit which pens, and of the zeal which pervades it—its style is elegant, and occasionally eloquent—the nature of his subject makes him sometimes obscure, and the peculiarities of his system leave him sometimes open to objection; but, were his enthusiasm for mere symptoms fairly balanced by an ordinary respect for morbid anatomy, we could have nothing, but the complicated minuteness of his analytical tables, to induce us to qualify in any way the very favourable estimate which we entertain of his taste and talents.

